# Mindfulness Trait Predicts Neurophysiological Reactivity Associated with Negativity Bias: An ERP Study

**DOI:** 10.1155/2015/212368

**Published:** 2015-06-01

**Authors:** Nerissa S. P. Ho, Delin Sun, Kin-Hung Ting, Chetwyn C. H. Chan, Tatia M. C. Lee

**Affiliations:** ^1^Laboratory of Neuropsychology, The University of Hong Kong, Pok Fu Lam, Hong Kong; ^2^Laboratory of Cognitive Affective Neuroscience, The University of Hong Kong, Pok Fu Lam, Hong Kong; ^3^Applied Cognitive Neuroscience Laboratory, The Hong Kong Polytechnic University, Hung Hom, Hong Kong; ^4^The State Key Laboratory of Brain and Cognitive Sciences, The University of Hong Kong, Pok Fu Lam, Hong Kong

## Abstract

This study explored the relationship of mindfulness trait with the early and late stages of affective processing, by examining the two corresponding ERP components, P2 and LPP, collected from twenty-two male Chinese participants with a wide range of meditation experiences. Multiple regression analyses was performed on the mindfulness scores, as measured by CAMS-R, with the subjective affective ratings and ERP data collected during an emotion processing task. The results showed that increased mindfulness scores predicted increased valence ratings of negative stimuli (less negative), as well as increased P2 amplitudes at the frontocentral location for positive compared to negative stimuli. Based on these findings, a plausible mechanism of mindfulness in reducing negativity bias was discussed. Moreover, our results replicated previous findings on the age-related increase of P2 amplitudes at the frontal sites for positive compared to neutral stimuli. Since the locations at which P2 amplitudes were found as associated with age and mindfulness differed, as did the emotional contents of the stimuli being compared, indicating that the effect of age did not confound our findings on mindfulness and the two factors might operate on early affective processing from distinct sources and mechanisms.

## 1. Introduction

Mindfulness is a disposition for describing people with a high level of self-regulated attention to be aware of the moment-to-moment flow of experiences, as well as an attitude for open, nonjudgmental, and receptive orientation. People high in mindfulness have good clarity and vividness of ongoing events, which frees them from being easily restricted to or preoccupied by distractions [1, 2]. It is widely acknowledged that mindfulness can be cultivated by mental training during meditation, a common practice in Buddhism and other contemplative traditions [3–5]. More importantly, there is mounting evidence that mindfulness can promote well-being [6, 7], as demonstrated by the high efficacy of mindfulness-based interventions on the treatment of psychological disorders in clinical studies [8, 9]. While well-being can be directly influenced by the processing of emotional information [10, 11], an emerging body of evidence has demonstrated that mindfulness can have a positive impact on affective processing [12, 13]. Hence, it is possible that the effect of mindfulness on well-being may be operated through its intertwined effect on affective processing. Nevertheless, neither the neural correlates of mindfulness nor its impacts on affective processing are well understood; therefore further investigation is required.

### 1.1. Mindfulness in Early Stage Processing

Several postulations have proposed that mindfulness may operate by modulating affective processing at different stages. One prominent view claimed that mindfulness could enhance attention for processing exogenous sensory information during the early stage of emotion generation to increase awareness of the ongoing events [14–16]. As stated by these theorists, the increase in bottom-up attention, when encompassed with an open, accepting, and compassionate attitude of mindfulness, would enable mindful people to better focus on the sensory aspects of their emotional encounters and detach from their habitual, reflexive emotional responses. This postulation on increased sensory attention combined with dampened emotional reactivity associated with mindfulness concurred with the findings of many past studies. For example, a longitudinal study showed that three months of intensive mindfulness meditation training increased perceptual sensitivity and improved attention vigilance [17]. Neuroimaging studies reported that mindfulness practitioners had enhanced sensory attention, as reflected by increased cortical thickness [18], and heightened neural activities in response to pain stimuli [19] which were observed in brain regions related to interoceptive and sensory processing, including the posterior insula and secondary somatosensory cortices. A study, based on event-related potential (ERP), also claimed that there was meditation-associated (suggesting mindfulness) increase in preattentive processing of automatic auditory change detection, by showing that meditators (compared to nonmeditators) immediately after (compared to before) concentrative meditation practices had significantly larger amplitudes of mismatch negativity (MMN, negativity around 100 to 250 ms after stimuli, an index for preattentive perceptual processing as measured by the amplitude difference between standard and deviant stimuli) using an auditory oddball task [20]. Besides enhanced attention, the effect of mindfulness on early stage affective processing was also reflected by the attenuation of emotional reactivity, as demonstrated by the reduction of emotional interference and skin conductance in response to negative pictures for the mindful participants [21] and the reduction of neurophysiological response to performance feedback indexed by the ERP component for feedback-related negativity (FRN, occurred approximately 250 ms after presentation of feedback, which was traditionally used for indexing response to aversive feedback, but was also found to arise as a positive response to rewards) [22]. Furthermore, enhanced perceptual clarity and decreased automatic reactivity were observed in meditation practitioners from a recent electroencephalography (EEG) study [23].

### 1.2. Mindfulness in Late Stage Processing

On the other hand, the effects of mindfulness reported on cognitive processing of emotional information during the later stage of emotion appraisal were more divergent. One view suggested that mindfulness could increase cognitive processing by expanding the metacognitive control and flexibility in shifting cognitive sets, hence facilitating disengagement of any previously conditioned appraisal for reconstrual of more positive ones, a process known as decentering or reperceiving that would help to engender hope and resilience [24]. Another group shared a similar view that mindfulness could increase exposure and reappraisal by enabling better recall of extinction learning, particularly for negative emotions [25]. As explained by these authors, mindfulness was associated with reduction of gray matter concentration in the amygdala (the brain region for emotion processing) [26] but also proliferation in the ventromedial prefrontal cortex (vmPFC) and hippocampus (brain regions for memory retrieval and cognitive control) [27, 28]; hence, the mechanism of mindfulness resembled those in the brain network associated with recall of memory extinction to signal extinguished context, which also showed increased emotional regulation of vmPFC and the hippocampus, together with downregulation of the amygdala [29]. A recent functional neuroimaging study demonstrated a similar finding and reported that mindfulness could reduce emotion processing (indicated by lower activation in the right amygdala) and increase cognitive regulation (indicated by higher activation in the dorsal medial PFC and other prefrontal regions) [30].

On the contrary, it was also claimed that mindfulness would inhibit, instead of increase, cognitive appraisal of affective information, since there were reduced activity observed in the executive and evaluative areas (including the prefrontal cortex and hippocampus) when meditation practitioners were stimulated by pain, while functional connectivity between executive and pain-related cortices was decreased [31].

### 1.3. The Present Study and Hypothesis

According to the above discussions, mindfulness would affect the attention/attentional reactivity and cognitive evaluation at two temporal stages of affective processing for emotional regulation. As claimed by the theorists, mindfulness would help to cultivate the ability for detaching from habitual reactivity, by also encompassing an open, accepting, and compassionate attitude. These mechanisms, particularly when applied to negative events, seemed to be contrasting with a phenomenon proposed by the evolutionists, known as attentional bias of threat or “negativity bias,” which was marked by more instantaneous and pronounced reactivity (including physiological, neural, and cognitive responses) upon encountering of negative (compared to neutral or positive) events [32, 33] due to the inherent nature of human beings in paying attention to threats so as to help them escape from dangers. Considering these postulations together, it seemed that the effect of mindfulness trait could counteract on the negativity bias effect, by detaching from the reflexive reactivity of negativity bias upon encountering of negative events. Hence, we studied the effect of mindfulness on two stages of affective processing by comparing with the ERP components that were frequently studied in past studies of negativity bias, that is, P2 and LPP [34–36].

Specifically, P2 is the positivity occurring around 200 ms, which is most prominently observed at the midline frontocentral sites. Although P2 was initially studied as an auditory evoked potential, the topography of P2 appeared to be similar across visual and somatosensory modalities. It has been suggested that P2 was nonhabituating and reflected the inhibition of interference from irrelevant stimuli during stimulus discrimination, with enhanced amplitudes in response to nontarget (compared to target) stimuli in oddball paradigms [see review: [37]]. Moreover, some past studies found P2 increased in both amplitude and latency with advancing age, particularly at the anterior sites [38, 39].

Alternatively, LPP is a midline, positive-going amplitude that is most evident at the centroparietal region starting at 300 ms after the presentation of the stimulus and lasting for several hundred milliseconds [40, 41]. LPP was thought to be associated with complex cognitive activities associated with subjective evaluation of stimuli, including their probability and meaning [42]. It has been suggested that more pronounced LPP would be elicited by emotional stimuli, because of their higher motivational relevance which would demand more cognitive evaluation [43, 44]. However, such emotional effect on LPP was nonhabituating in response to repeated stimuli [see review: [45]]. Moreover, it has been reported that negativity bias could be indexed by enlarged amplitudes of P2 for negative compared to positive events and enlarged amplitudes of LPP for negative compared to both neutral and positive events [34, 35].

In sum, the present study aimed to identify the neurophysiological correlates of mindfulness in people with meditation experience, by discerning the different temporal stages of affective processing in response to exogenous stimulation, using scalp-recorded event-related brain potentials (ERPs). Specifically, we explored the relationship of trait mindfulness with the temporal characteristics of P2 and LPP using linear regression analysis. Participants with various degrees of the trait mindfulness, as measured by self-report questionnaires, were presented with standardized pictures exhibiting pleasant, negative, and neutral negative scenes [46] while their scalp potentials were recorded. We hypothesized that trait mindfulness would predict P2 amplitude, the early index of negativity bias. In a recent behavioral study, mindfulness was found to be associated with reduced negativity bias stemming from increased positive judgment [47]. Hence, we further predicted that mindfulness would be associated with diminished negativity bias, that is, increased P2 amplitudes for positive compared to negative stimuli. On the other hand, we had no a priori hypothesis on the relationship of trait mindfulness with LPP because of the contradictory views of mindfulness on cognitive appraisal; however, the results obtained would add to the evidence on late evaluation theories of mindfulness.

## 2. Method

### 2.1. Participants

Chinese male adults with interest or experience in practicing meditation were recruited either from a Buddhist meditation network in Hong Kong or from the local community through advertisements posted on a university campus with the following selection criteria: (1) native Chinese speaker, male, between 40 and 70 years of age, with minimum education of secondary three level; and (2) healthy, with normal or corrected-to-normal vision and no history of neurological or memory diseases. The forms of meditation reported by these participants were based on the Theravāda school of Buddhism, either focused-attention meditation (*ānāpānasati*) or loving-kindness meditation (*mettā*). The information was also verified by the teacher of these meditators and by Venerable Jing Yin. Only men were included in the present study, so as to control for the gender-related effect on affective processing [48–50]. Twenty-two Chinese men with mean (range) age of 57.45 ± 6.43 (46 to 70) years and average (range) education of 14.77 ± 3.21 (9 to 20) years participated after being fully briefed on the study and gave their informed consent. The recruited group also had an average (range) of 6.52 ± 8.61 (<1 to 25) years of meditation experiences when they took part in the experiment. This allowed more homogeneity in terms of motivational preference, and yet there was enough diversity of dispositional mindfulness included in the group. The study was approved by the Institutional Review Board of Hong Kong Polytechnic University. All participants received a reimbursement of HKD $320 upon completion of the study as compensation for time and travel expenses.

### 2.2. Materials and Procedure

#### 2.2.1. Mindfulness and State Affect Measures

The Cognitive and Affective Mindfulness Scale Revised [CAMS-R, [51]] was administered to the participants to assess their mindfulness. CAMS-R is a brief and yet broad measure of mindfulness composed of 12 items that capture the four major components of the multifaceted conceptualization of mindfulness, namely, regulation of attention, orientation to present experience, awareness of experience, and acceptance/nonjudgment toward experience. Respondents are required to rate how often each item applies to their everyday approach to thoughts and feelings using a four-point Likert scale (1 = rarely/not at all, to 4 = almost always).

It has been suggested that state affect was associated with trait mindfulness and would also have an effect on affective processing [52]. To control for this potential confounding effect, the Chinese Affect Scale [CAS, [53]] for measuring the state affect was also administered. CAS is a validated Chinese version of the Positive and Negative Affect Schedule [PANAS, [54]] with 10 items in each of the two subscales for Positive Affect (PA) and Negative Affect (NA). Respondents are required to rate the extent to which they experience the 20 items of affect markers at the moment of response using a five-point Likert scale (1 = very slightly or not at all, to 5 = extremely).

#### 2.2.2. Stimulus Materials

Sixty full-color images from the International Affective Picture System [IAPS, [55]] with the highest valences and arousal ratings in published norms were selected. (The IAPS image numbers were as follows: positive: 1440, 1610, 1710, 1750, 1920, 2040, 2050, 2057, 2058, 2070, 2080, 2150, 2260, 2340, 2530, 2550, 5760, 5910, 8190, 8470; neutral: 1616, 2381, 2487, 2495, 2514, 2702, 2850, 2870, 5395, 5520, 5532, 5533, 5740, 6910, 7080, 7090, 7100, 7500, 7550, 7830; negative: 2141, 2205, 2800, 2900, 3220, 3230, 3301, 3350, 9050, 9140, 9181, 9220, 9410, 9421, 9520, 9560, 9571, 9910, 9911, 9921.) They were grouped into three categories (20 pictures in each category, with equal proportions of human and nonhuman content, excluding those with mutilation or erotic contents) by their emotional valence. The mean and standard deviation of the IAPS normative ratings for the three groups for valence (1 = very negative, to 9 = very positive) are positive = 8.01 ± 1.38, negative = 2.13 ± 1.47, and neutral  =  5.25 ± 1.40; and for arousal (1 = very not arousing, to 9 = very arousing) are positive = 4.68 ± 2.44, negative = 5.51 ± 2.17, and neutral = 3.45 ± 2.01. These picture stimuli were resized to a standard dimension of approximately 10 × 6 cm for presentation on a computer monitor with a visual angle of within 10 degrees.

#### 2.2.3. Experimental Task

**Figure 1:**
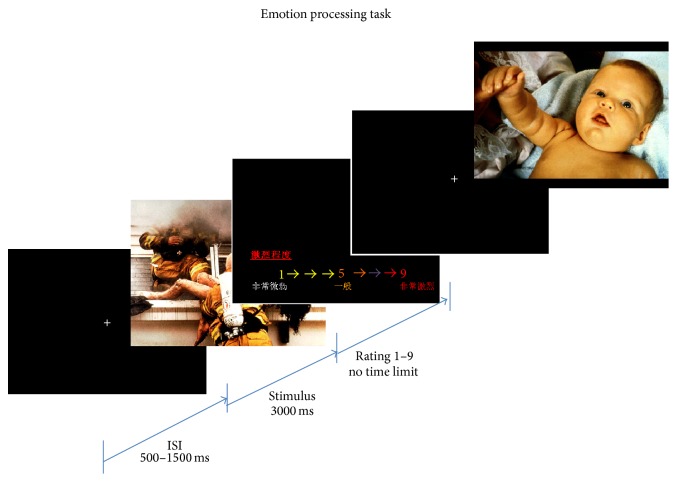
Schematic diagrams of emotion processing task.

At the beginning of each trial, participants were required to focus on a white central fixation-cross presented on a black screen, with durations varying from 500 to 1500 ms (in steps of 500 ms). Participants then passively viewed a three-second presentation of an image that was selected from the 60 IAPS pictures using a pseudorandom order so that consecutive presentations were not of the same image; however, consecutive presentation of picture stimuli with same valence was allowed, as valence of preceding stimuli or habituation effect was found to have insignificant effect on emotional ERP modulation based on past studies [41, 56]. Before the next trial, participants were required to rate the picture just presented on its valence or arousal (alternated by block) (see Figure 1).

Each block consisted of 60 trials, totaling 240 trials from four blocks for the entire task. These blocks requested ratings of arousal or valence alternately and counter-balanced in sequence across participants. Short breaks (one or two minutes) were offered at every 30-trial interval, and the average administration time was approximately 40 minutes. All stimulus presentations and rating responses were controlled by E-Prime software v1.1 (Psychology Software Tools, Pittsburgh, PA).

#### 2.2.4. Experimental Procedures

Participants were seated in a dimly lit, sound attenuated room to perform the emotion processing task for EEG recordings after they had filled out the self-report questionnaire. Before the experiment and after EEG preparations, participants were asked to practice meditation for 30 minutes, in order to align the state effect of meditation across the group, followed by a detailed explanation of the experimental instructions with 12 practice trials. Upon completion of the experimental task, participants were also requested to rate the clarity (1 = very unclear, to 9 = very clear) and stability (1 = very unstable, to 9 = very stable) of their mental states during the task.

### 2.3. ERP Data Acquisition and Preprocessing

Scalp electroencephalograms (EEGs) were recorded from a 64-channel elastic cap embedded with Ag-AgCl electrodes according to the extended 10–20 system, using a SynAmps2 amplifier, a Quikcap system, and Acquire 4.3 software (Compumedics Neuroscan, Charlotte, NC, USA). All channel data were recorded continuously at a sampling rate of 1000 Hz, with impedance maintained below 5 kΩ. All channels were referenced to the left mastoid. The ground electrode was positioned on the forehead. Vertical electrooculograms (EOGs) were recorded using electrodes located above and below the left eye, and horizontal EOGs were recorded from electrodes at the outer canthus of each eye.

The recorded EEG data were preprocessed offline using SPM8 software (Wellcome Trust Centre, UK). After being converted into SPM data format, the raw signals were band-pass filtered (0.5~30 Hz), corrected for eye artifacts [57], rereferenced to the computed average of the whole-scalp EEG channels, downsampled to 200 Hz, and then cut into epochs from −200 ms to 720 ms poststimulus onset. After baseline correction (−200~0 ms served as the baseline), epochs containing amplitudes exceeding ±100 *μ*V were removed (0 to 14.6% of epochs were rejected for all participants). Channels that had more than 20% of epochs removed would have been marked as bad channels and replaced by linear interpolation of the neighboring channels (none were marked as bad). Epochs for each participant's individual trials were aggregated using robust averaging [58] based on the stimulus type (positive, negative, and neutral).

### 2.4. Data Analysis

All data from self-reported measures, behavioral responses (task responses for the arousal and valence ratings of the IAPS pictures), and ERP results were analyzed by the R System for Statistical Computing version 3.1.1 (R Development Core Team, 2012). Since age has been reported as having significant effects on P2, this variable would be analyzed in parallel with CAMS-R so that their relationship with the outcome variables, if any, could be delineated.

For the behavioral data, we examined the relationship between the self-report measures (predictor variables: CAMS-R and age) and participants' responses on affective ratings (outcome variables: arousal and valence ratings of the IAPS pictures provided by the participants).

For the ERP data, firstly, we evaluate if there was emotional modulation effect (three categories of emotion were adopted for grouping the IAPS pictures using the published norms of their emotional valence) on the ERP components of P2 and LPP. Significant results would provide ground for the regression analysis in the next step, which examined the relationship between the self-report measures (predictor variables: CAMS-R and age) and the temporal characteristics of P2 and LPP evoked by pictures of three different emotional contents (outcome variables: contrasts of mean amplitudes among the three categories of emotional pictures and peak latencies of P2 and LPP evoked by among the three categories of emotional pictures).

All the regression analyses were conducted in two stages. First, the two predictors (age and CAMS-R) and the outcome variables were submitted to analysis by Pearson's correlation in order to provide an overview of the inter-correlations among them. Second, for those outcome variables found to have significant correlations with either or both of the predictors, multiple regression analyses were conducted to further evaluate the predictive power of the predictor (CAMS-R and/or age) on these outcome variables. This would be done by comparing the regression model including only the correlated predictor variable (CAMS-R or age) with a second model including the remaining predictor, and then with a third model that also included the state affect (CAS-PA for ERP elicited by positive or positive compared to neutral pictures, CAS-NA for negative or negative compared to neutral pictures, or both CAS-PA and CAS-NA for positive compared to negative pictures).

For the ERP data, time windows, and electrode sites were determined in reference to the previous literature and by visual inspection of the present data. Hence, time windows for P2 and LPP were defined as 140–280 ms and 400–700 ms, respectively. Electrode sites were defined as five midline channels at three locations of frontal (FZ, F1, F2, F3, and F4), frontocentral (FCZ, FC1, FC2, FC3, and FC4), and central (CZ, C1, C2, C3, and C4) for P2 and four locations of frontocentral (FCZ, FC1, FC2, FC3, and FC4), central (CZ, C1, C2, C3, and C4), centroparietal (CPZ, CP1, CP2, CP3, and CP4), and parietal (PZ, P1, P2, P3, and P4) regions for LPP.

Moreover, before correlation or regression analyses of the ERP data, we also examined whether there were any effects of emotion and/or location on the mean amplitudes of P2 and LPP by conducting a two-way repeated-measures analysis of variance (ANOVA) on each of the two ERP components separately, that is, 3 (emotions: positive, negative, and neutral) by 3 (locations: frontal, frontocentral and central) for P2 and 3 (emotions: positive, negative, and neutral) by 4 (locations: frontocentral, central, centroparietal, and parietal) for LPP.

Greenhouse-Geisser's method was adopted to correct for violations of sphericity, and False Discover Rate (FDR) and Bonferroni methods were used to correct for multiple comparisons in the Pearson correlations and post-hoc analyses, respectively.

## 3. Results

### 3.1. Descriptive Statistics for the Demographic Characteristics and Other Behavioral Variables

Means and standard deviations of the demographic characteristics, scores of self-report measures on mindfulness and state affect, and the affective ratings of the IAPS pictures are listed in Table 1.

One-way repeated-measures ANOVA on the arousal ratings of the IAPS pictures showed that they were not all the same for the three groups of pictures (*F*(2,42) = 52.902, *p* < .001). Post-hoc analysis further revealed that there were lower arousal ratings for positive than negative pictures (*F*(1,21) = 19.656, *p* < .001) and higher arousal ratings for positive than neutral (*F*(1,21) = 62.523, *p* < .001) and negative than neutral (*F*(1,21) = 70.931, *p* < .001) pictures.

### 3.2. Relationship between Self-Report Measures and Responses on Affective Ratings

#### 3.2.1. Correlations

Intercorrelations for age and CAMS-R and behavioral responses on both affective ratings are presented in Table 2. Only CAMS-R was found to be significantly correlated with valence rating of negative pictures (*r*(20) = .634, *p* = .007) and also marginally significant in correlating with valence ratings of positive pictures (*r*(20) = −.459, *p* = .081). Neither correlations for arousal rating with CAMS-R nor correlations for age with any affective ratings were found to be significant.

#### 3.2.2. Multiple Regressions

Since correlation results only found significant correlations of CAMS-R with valence ratings, multiple regression analyses were conducted to evaluate the predictive power of CAMS-R on valence ratings, and this was done separately on positive and negative pictures. Results of all analyses for comparing the three regression models varying from one to three predictors are presented in Table 3.

**Figure 2:**
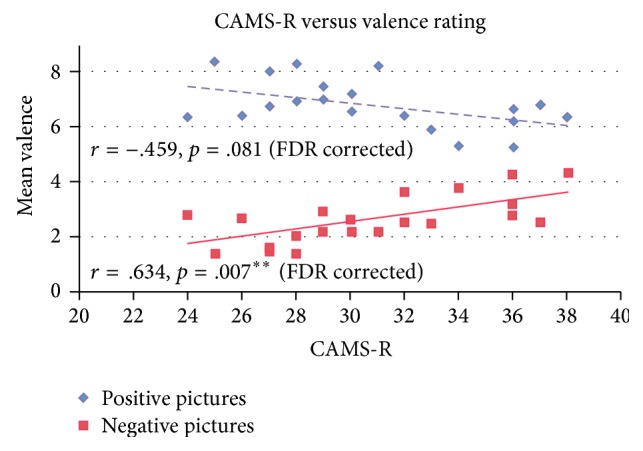
Scatterplot showing that CAMS-R negatively correlates with valence ratings of positive pictures (blue) and positively correlates with valence ratings of negative pictures (red).

For both positive and negative pictures, CAMS-R alone explained a significant amount of the variance in ratings of valence. When age or both age and state effect were added to the second or third model, changes in the variance of valence ratings explained by the models were not significant, as observed for both positive (Model 1 to 2: Δ*R* ^2^ = 6.12%, *F*(1,19) = 1.596, *p* = .222; Model 2 to 3: Δ*R* ^2^ = 7.23%, *F*(1,18) = 1.985, *p* = .176) and negative pictures (Model 1 to 2: Δ*R* ^2^ = .64%, *F*(1,19) = .206, *p* = .655; Model 2 to 3: Δ*R* ^2^ = 4.12%, *F*(1,18) = 1.350, *p* = .261). The results suggested that age and state affect offered little additional predictive power beyond that contributed by CAMS-R. Moreover, CAMS-R remained a significant predictor in all models for both types of pictures, confirming that CAMS-R was a significant predictor for the valence ratings of the pictures, even when the potential effects of age and state affect were considered. Increases in CAMS-R predicted lower valence ratings of positive pictures and higher valence ratings of negative pictures; see the scatterplot presented in Figure 2.

### 3.3. Emotion and Location Effects on ERP Data

#### 3.3.1. P2

Results of the two-way repeated-measures ANOVA (3 emotions × 3 locations) on the mean amplitude of P2 component showed that there were significant main effects of both emotions (*F*(2, 42) = 35.29, *p* < .001, *η*_*p*_^2^ = .627) and locations (*F*(2, 42) = 31.00, *p* < .001, *η*_*p*_^2^ = .596), whereas the interaction effect of emotions × locations was found to be insignificant (*F*(4, 84) = 1.19, *p* = ns, *η*_*p*_^2^ = .054).

Post-hoc analysis showed that P2 elicited by positive pictures was significantly more positive than P2 elicited by both negative (*F*(1, 21) = 53.54, *p* < .001, *η*_*p*_^2^ = .718) and neutral (*F*(1, 21) = 41.65, *p* < .001, *η*_*p*_^2^ = .665) pictures by collapsing all locations; no significant difference was observed for P2 elicited by negative pictures compared to neutral ones (*F*(1, 21) = .225, *p* = 1.000, *η*_*p*_^2^ = .010).

For location effect, post-hoc analysis showed that P2 increased significantly from anterior to more posterior locations, that is, from frontal to frontocentral sites (*F*(1, 21) = 29.60, *p* < .001, *η*_*p*_^2^ = .585) and from frontocentral to central sites (*F*(1, 21) = 26.16, *p* < .001, *η*_*p*_^2^ = .555) after collapsing for all emotions.

Furthermore, individual *t*-tests for P2 elicited by positive compared to both negative and neutral pictures at all locations showed that these results were all significant (*p* < .001 for frontocentral and central locations when compared to both emotions, *p* < .01 for the frontal location).

**Figure 3:**
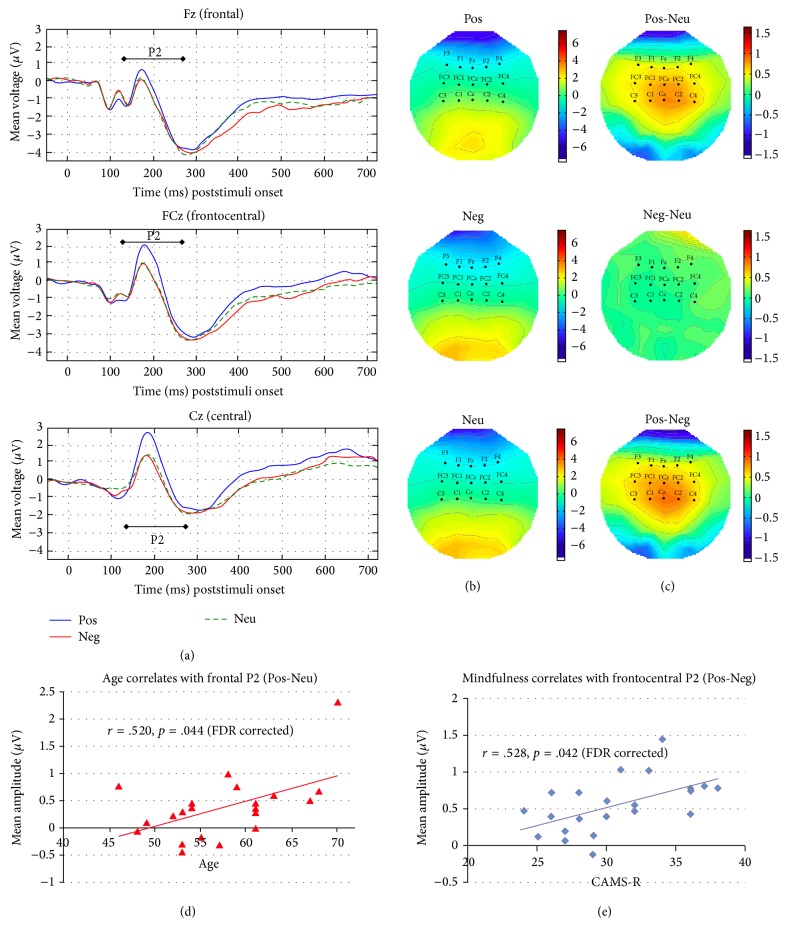
Waveforms and 2D topographies of P2. Waveforms elicited by presentation of IAPS pictures (positive, negative, and neutral) at representative midline electrodes (Fz, FCz, and Cz) are shown in (a). 2D topographies of the mean amplitudes averaged between 140 and 280 ms scalp distributions showing the original activities elicited by positive, negative, and neutral pictures (b) and differences between activities elicited by positive and neutral, negative and neutral, and positive and negative pictures (c) are shown. Scatterplot representing age positively correlates with difference of the mean amplitudes of P2 averaged between 140 to 280 ms as elicited by positive and neutral pictures collapsed across five channels at the frontal location (F1, F2, F3, F4, and Fz) is shown in (d). Scatterplot representing CAMS-R positively correlates with difference of the mean amplitudes of P2 averaged between 140 and 280 ms as elicited by positive and negative pictures collapsed across five channels at the frontocentral location (FC1, FC2, FC3, FC4, and FCz) are shown in (e). (Pos: positive; Neg: negative; Neu: neutral; Pos-Neu: positive-negative; Neg-Neu: negative-neutral; Pos-Neg: positive-negative; CAMS-R: Cognitive and Affective Mindfulness Scale Revised; IAPS: International Affective Picture System).

Figure 3 showed (a) the mean amplitude waveforms of P2 component (140–280 ms) elicited by presentation of IAPS pictures (positive, negative, and neutral) at representation midline electrodes (Fz, FCz, and Cz) and the 2D topographies of the (b) original and (c) contrasted activities averaged for the entire time window.

#### 3.3.2. LPP

Results of the two-way repeated-measures ANOVA (3 emotions × 4 locations) on the mean amplitude of LPP component also found significant main effects of emotions (*F*(2, 42) = 10.30, *p* < .001, *η*_*p*_^2^ = .329) and locations (*F*(3, 63) = 36.38, *p* < .001, *η*_*p*_^2^ = .634), as well as an interaction effect of emotions × locations (*F*(6, 126) = 7.03, *p* = .004, *η*_*p*_^2^ = .251).

Post-hoc analysis showed that LPP elicited by positive pictures was significantly more positive than LPP elicited both negative (*F*(1, 21) = 8.19, *p* = .027, *η*_*p*_^2^ = .281) and neutral (*F*(1, 21) = 14.18, *p* = .003, *η*_*p*_^2^ = .403) pictures when all locations were considered; LPP elicited by negative pictures compared to neutral ones was not significantly different, but only showed a marginal trend (*F*(1, 21) = 5.273, *p* = .096, *η*_*p*_^2^ = .201).

For location effect, post-hoc analysis also showed an increasing trend of LPP from anterior to posterior direction, with significant differences from frontocentral to central sites (*F*(1, 21) = 30.662, *p* < .001, *η*_*p*_^2^ = .594) and from central to parietocentral to central sites (*F*(1, 21) = 41.668, *p* < .001, *η*_*p*_^2^ = .665), but not from parietocentral to parietal sites (*F*(1, 21) = 4.932, *p* = ns) after collapsing all emotions.

Furthermore, individual *t*-tests for the four locations of LPP elicited by different pairs of emotions showed a significantly larger LPP for positive compared to negative pictures at both frontocentral (*p* < .001) and central locations (*p* < .01) but not at the other two posterior locations. Similar trend was observed for positive compared to neutral pictures, which were significantly larger at frontocentral (*p* < .001) and central locations (*p* < .001) but not at the other two posterior locations. However, comparing negative with neutral pictures, no significant effect was observed; there was only a trend for larger negative than neutral LPPs, with a significant difference observed at uncorrected *p* values, at the central (*p* = .036, uncorrected), centroparietal (*p* = .022, uncorrected), and parietal (*p* = .034, uncorrected) sites.

**Figure 4:**
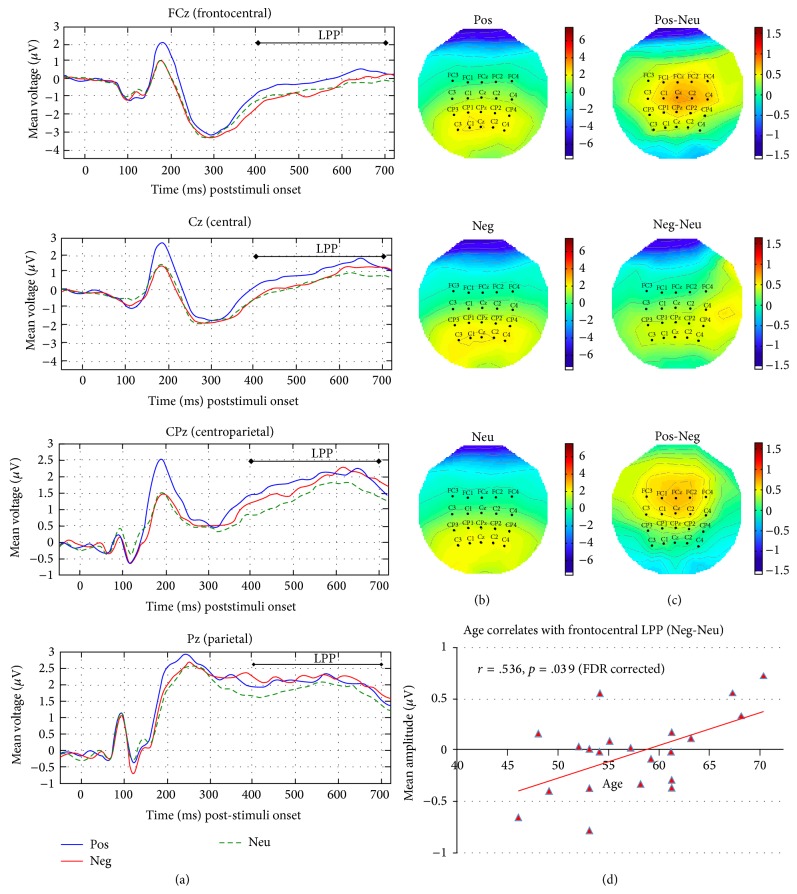
Waveforms and 2D topographies of LPP. Waveforms elicited by presentation of IAPS pictures (positive, negative, and neutral) at representative midline electrodes (CPz and Pz) are shown in (a). 2D topographies of the mean amplitudes averaged between 400 and 700 ms scalp distributions showing the original activities elicited by positive, negative, and neutral (b), and differences between activities elicited by positive and neutral, negative and neutral, and positive and negative pictures (c) are shown. Scatterplot representing age positively correlates with difference of the mean amplitudes of LPP averaged between 400 and 700 ms as elicited by negative and neutral pictures collapsed across five channels at the frontocentral location (FC1, FC2, FC3, FC4, and Fz) are shown in (d). (Pos: positive; Neg: negative; Neu: neutral; Pos-Neu: positive-negative; Neg-Neu: negative-neutral; Pos-Neg: positive-negative; CAMS-R: Cognitive and Affective Mindfulness Scale Revised; IAPS: International Affective Picture System).

Figure 4 shows (a) the mean amplitude waveform of the LPP component (400–700 ms) elicited by presentation of IAPS pictures (positive, negative, and neutral) at the represented midline electrodes (FCz, Cz, CPz, and Cz) and the 2D-topographies for (b) original and (c) contrasted activities averaged for the entire time window.

In sum, these results showed that emotional modulation effects were observed in both components of P2 and LPP, which might be associated with either or both the valence and arousal ratings as the three categories of pictures differed significantly in terms of both dimensions.

### 3.4. Relationship between Self-Report Measures and ERP Data

#### 3.4.1. Correlations

Table 4 presents the results of correlation between self-report measures (age and CAMS-R) and the two characteristics of P2 and LPP, namely (Table 4(a)), contrasts of mean amplitudes among the three pictures categories, including the differences between positive and neutral pictures (Pos-Neu), negative and neutral pictures (Neg-Neu), and positive and negative pictures (Pos-Neg), as well as (Table 4(b)) peak latencies of the original waveforms for positive, negative, and neutral pictures.

Two significant correlations were observed for P2. First one was between age and the frontal P2 mean amplitude contrast of positive and neutral pictures (Pos-Neu: *r*(20) = .520, *p* = .044). Second was between CAMS-R and the frontocentral P2 mean amplitude contrast of positive and negative pictures (Pos-Neg: *r*(20) = .528, *p* = .042). For LPP, the only significant correlation observed was between age and the frontocentral LPP mean amplitudes contrast of negative and neutral pictures (Neg-Neu: *r*(20) = .536, *p* = .039).

However, no significant correlations were observed for peak latencies for both components at all locations and for all emotions.

#### 3.4.2. Multiple Regression

Based on the correlation results, multiple regression analyses were conducted to evaluate the predictive power of age and/or CAMS-R on the three corresponding P2 and LPP mean amplitude contrasts for which significant correlations were found. Analysis results for the three regression models varying from one to three predictors are presented in Table 5.

For the frontal P2 mean amplitude contrast of positive to neutral pictures (Pos-Neu), age alone explained a significant amount of the variance (*R* ^2^ = 27.01%, *p* = .013). Addition of a second (CAMS-R) and third predictors (CAS-PA) would not significantly increase the variance explained (Model 1 to Model 2: Δ*R* ^2^ = 8.89%, *F*(1,19) = 2.637, *p* = .121; Model 2 to Model 3: Δ*R* ^2^ = .04%, *F*(1,18) = .011, *p* = .918).

For the frontocentral P2 mean amplitude contrast of positive to negative pictures (Pos-Neg), CAMS-R alone explained a significant amount of the variance (*R* ^2^ = 27.87%, *p* = .012). Addition of a second (age), third, and fourth predictors (CAS-PA and CAS-NA) would not significantly increase the variance explained (Model 1 to Model 2: Δ*R* ^2^ = .36%, *F*(1,19) = .096, *p* = .761; Model 2 to Model 3: Δ*R* ^2^ = 10.82%, *F*(2,17) = 1.508, *p* = .250).

For the frontocentral LPP mean amplitude contrast of negative to neutral pictures (Neg-Neu), again, age alone explained a significant amount of the variance (*R* ^2^ = 28.75%, *p* = .010). Addition of a second (CAMS-R) and third predictors (CAS-NA) would not significantly increase the variance explained (Model 1 to Model 2: Δ*R* ^2^ = .28%, *F*(1,19) = .076, *p* = .786; Model 2 to Model 3: Δ*R* ^2^ = .20%, *F*(1,18) = .051, *p* = .825).

All these results suggested that the second and third (or fourth) predictors offered little additional predictive power beyond that contributed by the primary predictor. Moreover, the primary predictors (age or CAMS-R) remained the significant predictors in all models of these analyses even when the effects of the potential confounding variables were considered. In sum, increased age predicted increased frontal P2 mean amplitude contrast of positive to neutral pictures (Pos-Neu) (see Figure 3(d)) and increased frontocentral LPP mean amplitude contrast of negative to neutral pictures (Neg-Neu) (see Figure 4(d)), whereas increased CAMS-R predicted increased frontocentral P2 mean amplitude contrast of positive to negative pictures (Pos-Neg) (see Figure 3(e)).

### 3.5. Additional Analysis on the Potential Influence by Arousal Ratings on ERP Data

Since the three groups of pictures were significantly differed, not only on emotional contents, but also the arousal ratings (based on the participants' response), the following additional analyses were performed to evaluate the potential effect of the arousal ratings by incorporating the differences of arousal ratings for the related contrast into the final models for verification.

For the frontal P2 mean amplitude contrast of positive to neutral pictures (Pos-Neu), addition of arousal ratings difference (Pos-Neu) would not significantly increase the variance explained (Δ*R* ^2^ = 3.37%, *F*(1,19) = .920, *p* = .350). Similarly, for the frontocentral P2 mean amplitude contrast of positive to negative pictures (Pos-Neg), addition of arousal ratings difference (Pos-Neg) would not significantly increase the variance explained (Δ*R* ^2^ = .29%, *F*(1,19) = .077, *p* = .784). While for the frontocentral LPP mean amplitude contrast of negative to neutral pictures (Neg-Neu), addition of arousal ratings difference (Neg-Neu) also would not significantly increase the variance explained (Δ*R* ^2^ = 4.78%, *F*(1,19) = 1.367, *p* = .257).

## 4. Discussion

The present study explored the relationships between trait mindfulness and the temporal characteristics of P2 and LPP, ERP components associated with emotional bias, and regulation, as elicited by different categories of IAPS pictures with different emotional contents. By using multiple regression analyses, these relationships were evaluated, by testing the predictive power of trait mindfulness on the mean amplitudes (for the various contrasts comparing different categories of the pictures) and peak latencies (for each category of the pictures) of the ERP components. The relationships between trait mindfulness and the self-reported arousal and valence ratings for the same set of IAPS pictures were also investigated. Throughout the analyses, age was included as an additional predictor variable to delineate this factor from the effect of mindfulness since past studies consistently reported the age-related effect of P2. State affect was also included in the regression models to avoid possible confounding effects.

### 4.1. Mindfulness on Early Stage Processing

The results confirmed our first a priori hypothesis that trait mindfulness predicted P2 mean amplitudes, but only for the contrast comparing positive and negative pictures at the frontocentral site. According to the past studies, negativity bias was associated with increased mean amplitudes of P2 on negative compared to positive events [34–36]. While these studies interpreted increased P2 amplitudes as enhanced attention to negative events, early P2 studies found that greater P2 amplitudes were associated with nontarget (compared to target) stimuli in auditory oddball paradigms [59, 60] and suggested that increased P2 amplitudes should be used to index lowered attention to nontarget stimuli since increased P2 would reflect increased processing of top-down inhibition to suppress interference of irrelevant stimuli and to drive selective attention towards target stimuli at a later phase [see the review in [37]]. In line with the second view, another study using a visual search paradigm also found that increased P2 amplitudes were associated with greater effort for suppressing irrelevant stimuli, by examining the factors that would lead to engagement of less efficient strategies (and thus greater effort) for target searching, including the number of distractors and other distractor features (e.g., the target-distractor similarity) [61].

Thus, by adopting the second view of P2 amplitude as index of interference suppression to reduce attention on nontarget stimuli, the present finding of positive relationship between mindfulness and P2 amplitudes on positive relative to negative pictures suggested that mindfulness could be associated with relative increase in effort to suppress attention on positive stimuli (i.e., a relative decrease in effort to suppress attention on negative stimuli). Compared to past studies of negativity bias, which found positive relationship of increased P2 amplitudes for negative relative to positive events (i.e., a relative increase in effort to suppress attention on negative stimuli), our results showed a reversed pattern of relative P2 amplitudes between positive and negative stimuli associated with mindfulness and negativity bias. This demonstrated that mindfulness trait could reduce effort to suppress attention on negative events associated with negativity bias, providing consistent neurophysiological evidence in support of the behavioral findings in a previous study reporting that mindfulness might reduce negativity bias [47].

Such negative relationship between negativity bias and mindfulness could be explained by the mechanism proposed in the early stage processing theory of mindfulness [14–16]. Based on these postulations, ability to detach from habitual reactivity could be enhanced by increased attention encompassed with an open, accepting, and compassionate attitude associated with mindfulness; hence, with the reported increase in relative attention of negative pictures (suggested by the decreased effort of suppression indexed by P2 amplitudes), increased mindfulness would increase the ability to detach from the habitual reactivity of negative stimuli and (i.e., instantaneous avoidance), therefore, reduce negativity bias.

Interestingly, the behavioral findings of the current study also showed that mindfulness only predicted valence ratings, particularly on negative pictures, but not arousal ratings. While valence was related to the classification of stimulus by their emotional content, arousal was associated with the intensity of processing. Taking together, our findings on the predictive power of mindfulness on both valence ratings and the P2 amplitudes on the contrast of positive to negative stimuli (Pos-Neg) (even when the effect of arousal was excluded) supported the role of mindfulness in stimulus classification and the interpretation of P2 as an index of effort to suppress interference for stimulus discrimination, which in turn, explained the early effect of mindfulness on and affective processing, particularly in reducing negativity bias.

Moreover, because of the close relationship between mindfulness and meditation and the fact that our participants were all meditators, it would be worth noting that our results were also consistent with previous meditation studies showing the association of enhanced positive and reduced negative emotions with meditation practice [62–64].

Lastly, replicating previous findings on the age-related effect of P2 [37, 65, 66], our regression analysis results also showed that age predicted P2 amplitudes at frontal sites. More interestingly, our results delineated the effect of age from the effect of mindfulness on P2 amplitudes: age mainly affected positive (compared to neutral) stimuli (Pos-Neu) at frontal locations, while mindfulness influenced the difference between positive and negative stimuli (Pos-Neg) at frontocentral sites (less anterior). These findings demonstrated that the relationship between P2 and mindfulness was not confounded by the effect of age, and they also reflected the multidimensional nature of P2, possibly from different sources and with different underpinning mechanisms operating at different locations. Further study will be required to explore the mechanisms underpinnings these differences.

### 4.2. Mindfulness in the Late Processing Stage

On the other hand, our result did not show any relationship of mindfulness trait with LPP, supporting neither the “positive reappraisal” nor “nonappraisal” view for the late evaluation theories of mindfulness. This was in contrast with a previous study [67] reporting that the enhancement effect of negative (versus positive/neutral) stimuli on LPP amplitudes during the late affective processing was controlled by meditation practices (which cultivate mindfulness) while no early stage effect was observed. One plausible explanation for the lack of LPP effect in our findings was insufficient arousal generated from the IAPS pictures we engaged. This factor could become particularly significant since emotional reactivity elicited by the stimuli had already been downregulated during the early stage processing (as in our case), leaving the remaining impact became unobservable on the late component.

Interestingly, we also observed that increased age predicted increased frontocentral LPP for the contrast for negative and neutral pictures. Further exploration would be required to explain this age-related effect of LPP and was outside the scope of the current study.

### 4.3. Limitations of the Present Study

There were several limitations in the present study. Firstly, only males with meditation experience were recruited, which will avoid potential confounds on affective processing by the effects of gender [48–50] and meditation [30, 68]; however, it also limits the generalization of the findings.

Secondly, although significant main effects of emotion and location were detected for both P2 and LPP, only positive pictures had significantly larger P2 and LPP than both negative and neutral pictures, but no significant difference was observed between negative and neutral pictures. Although similar “positivity only” results were also reported in a recent mindfulness study [22], this less common positivity effect might also be attributed to our sample of participants, who were targeted for inclusion of a wide variety of mindfulness traits while also controlling for mood state and motivational preference. Specifically, the participants were all meditation practitioners representing middle/late adulthood to the elderly. The combined effects of meditation training, which would help to foster positive emotion [68], together with the age-related positivity bias [69, 70] might have caused greater motivational relevance of positive emotions. Therefore, further research should investigate whether the findings could be generalized to female and younger populations, as well as those that have no experience with meditation.

Finally, as discussed above in the discussion on LPP, the IAPS pictures adopted for eliciting emotional response were only of medium arousal level which might not be strong enough to survive after regulation by the early stage processing to produce the potential effect on LPP. Future research should consider engagement of stimuli that can provoke stronger emotional arousal.

## 5. Conclusions

The present study found significant predictive power of mindfulness on P2 at the frontocentral location. Moreover, this effect of mindfulness was found to be distinguishable from the effect of age. More importantly, by combining these results with the prevailing theories of mindfulness and theories about negativity bias, we proposed a plausible mechanism by which mindfulness could reduce the effect of negativity bias. The findings help extend the current knowledge on how mindfulness operates on early stage affective processing to explain its salutatory effect on emotion regulation. Cultivation of the mindfulness trait might help to reduce the habitual stimulus-driven reactivity, especially in encountering negative events. This finding has significant implications for public health programs for people suffering from clinical or subclinical mood- and/or anxiety-related symptoms characterized by negativity bias in processing affective stimuli.

## Supplementary Material

Similar correlations as presented in Table 4(a), by using the original P2 and LPP waveforms for the three categories of pictures rather than the contrasts among them, were also calculated for reference. However, no significant result was found.

## Figures and Tables

**Table 1:** Descriptive statistics (mean ± SD) for demographics characteristics, mindfulness scores (CAMS-R and the four factors of attention, present-focus, awareness, and acceptance), state affect (CAS-PA and CAS-NA), and arousal and valence ratings of the IAPS pictures.

Demographics	Age	Years of education		
	57.45 ± 6.43	14.77 ± 3.21		

Mindfulness	CAMS-R	Years of meditation		
	30.64 ± 4.18	6.52 ± 8.61		
	CAMS-R factors			
	Attention	Present focus	Awareness	Acceptance
	8.05 ± 1.68	7.82 ± 1.62	6.95 ± 1.59	7.82 ± 1.62

State affect	CAS-PA	CAS-NA		
	23.91 ± 4.03	10.00 ± 5.92		

IAPS arousal ratings	Positive	Negative	Neutral	
	5.49 ± 1.71	6.74 ± 1.13	3.79 ± 1.57	

IAPS valence ratings	Positive	Negative	Neutral	
	6.79 ± 0.87	2.61 ± 0.83	5.16 ± 0.33	

*Note.* CAMS-R: Cognitive and Affective Mindfulness Scale Revised; CAS: Chinese Affect Scale; PA: Positive Affect; NA: Negative affect.

**Table 2:** Intercorrelations (*r*) for age, mindfulness (CAMS-R), and responses on arousal and valence ratings of the IAPS pictures.

Measures	(1) Age	(2) CAMS-R	(3) Arousal: positive	(4) Arousal: negative	(5) Arousal: neutral	(6) Valence: positive	(7) Valence: negative	(8) Valence: neutral
(1) Age	1							
(2) CAMS-R	.107	1						
(3) Arousal: positive	.210	−.081	1					
(4) Arousal: negative	−.024	−.386	.635	1				
(5) Arousal: neutral	.282	.157	.815^*∗∗∗*^	.294	1			
(6) Valence: positive	.197	−.459	.515	.661^*∗*^	.370	1		
(7) Valence: negative	−.011	.634∗∗	−.399	−.804^*∗∗∗*^	−.102	−.787^*∗∗*^	1	
(8) Valence: neutral	.352	−.066	.471	.356	.570	.577	−.348	1

*Note.* CAMS-R: Cognitive and Affective Mindfulness Scale Revised.

^*∗*^ *p* < .05, ^*∗∗*^ *p* < .01, and ^*∗∗∗*^ *p* < .001 (FDR-corrected).

**Table 3:** Results for multiple regression analysis for response on the affective ratings (only those that have significant correlations with age/CAMS-R were shown).

Valence	Positive picture				Negative pictures			
Ratings	*R* ^2^	*F*	*β*	*p*	*R* ^2^	*F*	*β*	*p*
Model 1	**21.05%**	**5.333**		.032^*∗*^	**40.25%**	**13.47**		.002^*∗∗*^
CAMR-S			−.459	.032^*∗*^			.634	.002^*∗∗*^
Model 2	27.17%	3.544		.049^*∗*^	40.89%	6.572		.007^*∗∗*^
CAMR-S			−.486	.002^*∗*^			.643	.002^*∗∗*^
Age			.249	.222			−.080	.655
Model 3	34.41%	3.147		.051	45.01%	4.912		.012^*∗*^
CAMR-S			−.539	.013^*∗*^			.531	.016^*∗*^
Age			.260	.193			−.105	.562
CAS-PA			.274	.176			—	—
CAS-NA			—	—			.531	.016^*∗*^

*Note.* CAMS-R: Cognitive and Affective Mindfulness Scale Revised; CAS: Chinese Affect Scale; PA: Positive Affect; NA: Negative affect. Rows highlighted denote the final selected models.

^*∗*^ *p* < .05 and ^*∗∗*^ *p* < .01 (uncorrected).

**(a):** 

	P2			LPP		
	Pos-Neu	Neg-Neu	Pos-Neg	Pos-Neu	Neg-Neu	Pos-Neg
	P2-frontal			LPP-frontocentral		
Age	.520∗	.174	.484	.443	.536∗	−.057
CAMS-R	.352	.033	.389	.193	.111	.095

	P2-frontocentral			LPP-central		
Age	.424	.444	−.003	.232	.410	−.103
CAMS-R	.277	−.240	-.528∗	.225	.029	.232

	P2-central			LPP-centroparietal		
Age	.222	.324	−.106	.035	.096	−.060
CAMS-R	.241	−.226	.458	.115	−.005	.208

				LPP-parietal		
Age				−.084	−.099	.007
CAMS-R				.078	−.002	.155

**(b):** 

	Pos	Neg	Neu	Pos	Neg	Neu
	P2-frontal			LPP-frontocentral		
Age	.246	.321	.341	.099	.591	.634
CAMS-R	−.175	−.147	.161	.591	.629	.591

	P2-frontocentral			LPP-central		
Age	−.004	.053	.191	−.403	.067	.144
CAMS-R	−.006	.163	.063	.216	.232	.229

	P2-central			LPP-centroparietal		
Age	.035	.044	.146	−.229	−.123	−.005
CAMS-R	.127	−.031	.052	.123	.136	.119

				LPP-parietal		
Age				−.013	−.217	−.003
CAMS-R				.053	.029	−.084

Note. Pos = positive; Neg = negative; Neu = neutral; Pos-Neu = positive-negative; Neg-Neu = negative-neutral; Pos-Neg = positive-negative; CAMS-R = Cognitive and Affective Mindfulness Scale Revised.

^*∗*^ *p* < .05 (FDR-corrected).

**Table 5:** Results for multiple regression analysis of ERP Data (only those with significant correlations with age/CAMS-R are shown).

	*R* ^2^	*F*	*β*	*p*
Frontal P2 (Pos-Neu)				
Model 1	**27.01%**	**7.403**		.013^*∗*^
Age			.520	.013^*∗*^
CAMR-S			—	—
Model 2	35.91%	5.323		.015^*∗*^
Age			.488	.016^*∗*^
CAMR-S			.300	.121
Model 3	35.95%	3.367		.042^*∗*^
Age			.488	.019^*∗*^
CAMR-S			.296	.143
CAS-PA			.020	.918
CAS-NA			—	—

Frontocentral P2 (Pos-Neg)				
Model 1	**27.87**%	**7.726**		.012^**∗**^
Age			—	—
CAMR-S			.528	.012^*∗*^
Model 2	28.23%	3.736		.043^*∗*^
Age			−.060	.761
CAMR-S			.534	.013^*∗*^
Model 3	39.04%	2.722		.064
Age			−.024	.903
CAMR-S			.726	.005^*∗∗*^
CAS-PA			−.054	.783
CAS-NA			.380	.101

Frontocentral LPP (Neg-Neu)				
Model 1	**28.75%**	**8.069**		.010^*∗*^
Age			.536	.010^*∗*^
CAMR-S			—	—
Model 2	29.03%	3.886		.038^*∗*^
Age			.536	.013^*∗*^
CAMR-S			.054	.786
Model 3	29.32%	2.478		.094
Age			.525	.018^*∗*^
CAMR-S			.029	.900
CAS-NA			−.051	.825

Note. Pos = positive; Neg = negative; Neu = neutral; Pos-Neu = positive-negative; Neg-Neu = negative-neutral; Pos-Neg = positive-negative; CAMS-R = Cognitive and Affective Mindfulness Scale Revised; CAS = Chinese Affect Scale; PA = Positive Affect; NA = Negative affect.

^*∗*^ *p* < .05 and ^*∗∗*^ *p* < .01 (uncorrected); rows highlighted denote the final selected models.

## References

[1] Brown K. W., Ryan R. M. (2003). The benefits of being present: mindfulness and its role in psychological well-being. *Journal of Personality and Social Psychology*.

[2] Bishop S. R., Lau M., Shapiro S. (2004). Mindfulness: a proposed operational definition. *Clinical Psychology: Science and Practice*.

[3] Salzberg S. (1995). *Loving-Kindness: The Revolutionary Art of Happiness*.

[4] Kabat-Zinn J. (1994). *Wherever You Go, There You Are: Mindfulness Meditation in Everyday Life*.

[5] Segall S. R. (2003). *Encountering Buddhism: Western Psychology and Buddhist Teachings*.

[6] Shapiro S. L., Oman D., Thoresen C. E., Plante T. G., Flinders T. (2008). Cultivating mindfulness: effects on well-being. *Journal of Clinical Psychology*.

[7] Ryan R. M., Huta V., Deci E. L. (2008). Living well: a self-determination theory perspective on eudaimonia. *Journal of Happiness Studies*.

[8] Segal Z. V., Williams J. M. G., Teasdale J. D. (2012). *Mindfulness-Based Cognitive Therapy for Depression*.

[9] Baer R. A. (2003). Mindfulness training as a clinical intervention: a conceptual and empirical review. *Clinical Psychology: Science and Practice*.

[10] Schwarz N., Clore G. L. (1983). Mood, misattribution, and judgments of well-being: informative and directive functions of affective states. *Journal of Personality and Social Psychology*.

[11] Gross J. J., John O. P. (2003). Individual differences in two emotion regulation processes: implications for affect, relationships, and well-being. *Journal of Personality and Social Psychology*.

[12] Jha A. P., Stanley E. A., Kiyonaga A., Wong L., Gelfand L. (2010). Examining the protective effects of mindfulness training on working memory capacity and affective experience. *Emotion*.

[13] Farb N. A. S., Anderson A. K., Mayberg H., Bean J., McKeon D., Segal Z. V. (2010). Minding one’s emotions: mindfulness training alters the neural expression of sadness. *Emotion*.

[14] Shapiro S. L., Carlson L. E., Astin J. A., Freedman B. (2006). Mechanisms of mindfulness. *Journal of Clinical Psychology*.

[15] Arch J. J., Craske M. G. (2006). Mechanisms of mindfulness: emotion regulation following a focused breathing induction. *Behaviour Research and Therapy*.

[16] Brown K. W., Ryan R. M., Creswell J. D. (2007). Mindfulness: theoretical foundations and evidence for its salutary effects. *Psychological Inquiry*.

[17] MacLean K. A., Ferrer E., Aichele S. R. (2010). Intensive meditation training improves perceptual discrimination and sustained attention. *Psychological Science*.

[18] Lazar S. W., Kerr C. E., Wasserman R. H. (2005). Meditation experience is associated with increased cortical thickness. *NeuroReport*.

[19] Gard T., Hölzel B. K., Sack A. T. (2012). Pain attenuation through mindfulness is associated with decreased cognitive control and increased sensory processing in the brain. *Cerebral Cortex*.

[20] Srinivasan N., Baijal S. (2007). Concentrative meditation enhances preattentive processing: a mismatch negativity study. *NeuroReport*.

[21] Ortner C. N. M., Kilner S. J., Zelazo P. D. (2007). Mindfulness meditation and reduced emotional interference on a cognitive task. *Motivation and Emotion*.

[22] Teper R., Inzlicht M. (2014). Mindful acceptance dampens neuroaffective reactions to external and rewarding performance feedback. *Emotion*.

[23] Cahn B. R., Delorme A., Polich J. (2013). Event-related delta, theta, alpha and gamma correlates to auditory oddball processing during vipassana meditation. *Social Cognitive and Affective Neuroscience*.

[24] Garland E., Gaylord S., Park J. (2009). The role of mindfulness in positive reappraisal. *Explore: The Journal of Science and Healing*.

[25] Hölzel B. K., Lazar S. W., Gard T., Schuman-Olivier Z., Vago D. R., Ott U. (2011). How does mindfulness meditation work? Proposing mechanisms of action from a conceptual and neural perspective. *Perspectives on Psychological Science*.

[26] Hölzel B. K., Carmody J., Evans K. C. (2010). Stress reduction correlates with structural changes in the amygdala. *Social Cognitive and Affective Neuroscience*.

[27] Hölzel B. K., Ott U., Gard T. (2008). Investigation of mindfulness meditation practitioners with voxel-based morphometry. *Social Cognitive and Affective Neuroscience*.

[28] Luders E., Toga A. W., Lepore N., Gaser C. (2009). The underlying anatomical correlates of long-term meditation: larger hippocampal and frontal volumes of gray matter. *NeuroImage*.

[29] Milad M. R., Wright C. I., Orr S. P., Pitman R. K., Quirk G. J., Rauch S. L. (2007). Recall of fear extinction in humans activates the ventromedial prefrontal cortex and hippocampus in concert. *Biological Psychiatry*.

[30] Lutz J., Herwig U., Opialla S. (2014). Mindfulness and emotion regulation—an fMRI study. *Social Cognitive and Affective Neuroscience*.

[31] Zeidan F., Martucci K. T., Kraft R. A., Gordon N. S., Mchaffie J. G., Coghill R. C. (2011). Brain mechanisms supporting the modulation of pain by mindfulness meditation. *The Journal of Neuroscience*.

[32] Cacioppo J. T., Gardner W. L. (1999). Emotion. *Annual Review of Psychology*.

[33] Northoff G., Richter A., Gessner M. (2000). Functional dissociation between medial and lateral prefrontal cortical spatiotemporal activation in negative and positive emotions: a combined fMRI/MEG study. *Cerebral Cortex*.

[34] Huang Y.-X., Luo Y.-J. (2006). Temporal course of emotional negativity bias: an ERP study. *Neuroscience Letters*.

[35] Carretié L., Martín-Loeches M., Hinojosa J. A., Mercado F. (2001). Emotion and attention interaction studied through event-related potentials. *Journal of Cognitive Neuroscience*.

[36] Ito T. A., Larsen J. T., Smith N. K., Cacioppo J. T. (1998). Negative information weighs more heavily on the brain: the negativity bias in evaluative categorizations. *Journal of Personality and Social Psychology*.

[37] Crowley K. E., Colrain I. M. (2004). A review of the evidence for P2 being an independent component process: age, sleep and modality. *Clinical Neurophysiology*.

[38] Friedman D., Simpson G., Hamberger M. (1993). Age-related changes in scalp topography to novel and target stimuli. *Psychophysiology*.

[39] Anderer P., Semlitsch H. V., Saletu B. (1996). Multichannel auditory event-related brain potentials: effects of normal aging on the scalp distribution of N1, P2, N2 and P300 latencies and amplitudes. *Electroencephalography and Clinical Neurophysiology*.

[40] Schupp H. T., Junghöfer M., Weike A. I., Hamm A. O. (2004). The selective processing of briefly presented affective pictures: an ERP analysis. *Psychophysiology*.

[41] Schupp H. T., Schmälzle R., Flaisch T., Weike A. I., Hamm A. O. (2012). Affective picture processing as a function of preceding picture valence: an ERP analysis. *Biological Psychology*.

[42] Johnson R. (1993). On the neural generators of the P300 component of the event-related potential. *Psychophysiology*.

[43] Cuthbert B. N., Schupp H. T., Bradley M. M., Birbaumer N., Lang P. J. (2000). Brain potentials in affective picture processing: covariation with autonomic arousal and affective report. *Biological Psychology*.

[44] Schupp H. T., Cuthbert B. N., Bradley M. M., Cacioppo J. T., Tiffany I., Lang P. J. (2000). Affective picture processing: the late positive potential is modulated by motivational relevance. *Psychophysiology*.

[45] Hajcak G., Macnamara A., Olvet D. M. (2010). Event-related potentials, emotion, and emotion regulation: an integrative review. *Developmental Neuropsychology*.

[46] Bradley M. M., Lang P. J., Coan J. A., Allen J. J. B. (2007). The International Affective Picture System (IAPS) in the study of emotion and attention. *Handbook of Emotion Elicitation and Assessment. Series in Affective Science*.

[47] Kiken L. G., Shook N. J. (2011). Looking up: mindfulness increases positive judgments and reduces negativity bias. *Social Psychological and Personality Science*.

[48] Han S., Fan Y., Mao L. (2008). Gender difference in empathy for pain: an electrophysiological investigation. *Brain Research*.

[49] Kring A. M., Gordon A. H. (1998). Sex differences in emotion: expression, experience, and physiology. *Journal of Personality and Social Psychology*.

[50] Singer T., Seymour B., O'Doherty J. P., Stephan K. E., Dolan R. J., Frith C. D. (2006). Empathic neural responses are modulated by the perceived fairness of others. *Nature*.

[51] Feldman G., Hayes A., Kumar S., Greeson J., Laurenceau J.-P. (2007). Mindfulness and emotion regulation: the development and initial validation of the Cognitive and Affective Mindfulness Scale-Revised (CAMS-R). *Journal of Psychopathology and Behavioral Assessment*.

[52] Kiken L. G., Shook N. J. (2014). Does mindfulness attenuate thoughts emphasizing negativity, but not positivity?. *Journal of Research in Personality*.

[53] Hamid P. N., Cheng S.-T. (1996). The development and validation of an index of emotional disposition and mood state: the Chinese affect scale. *Educational and Psychological Measurement*.

[54] Watson D., Clark L. A., Tellegen A. (1988). Development and validation of brief measures of positive and negative affect: the PANAS scales. *Journal of Personality and Social Psychology*.

[55] Lang P. J., Bradley M. M., Cuthbert B. N. (1999). *International Affective Picture System (IAPS): Technical Manual and Affective Ratings*.

[56] Schupp H. T., Stockburger J., Codispoti M., Junghöfer M., Weike A. I., Hamm A. O. (2006). Stimulus novelty and emotion perception: the near absence of habituation in the visual cortex. *NeuroReport*.

[57] Berg P., Scherg M. (1994). A multiple source approach to the correction of eye artifacts. *Electroencephalography and Clinical Neurophysiology*.

[58] Wager T. D., Keller M. C., Lacey S. C., Jonides J. (2005). Increased sensitivity in neuroimaging analyses using robust regression. *NeuroImage*.

[59] Novak G., Ritter W., Vaughan H. G. (1992). Mismatch detection and the latency of temporal judgments. *Psychophysiology*.

[60] García-Larrea L., Lukaszewicz A.-C., Mauguiere F. (1992). Revisiting the oddball paradigm. Non-target vs neutral stimuli and the evaluation of ERP attentional effects. *Neuropsychologia*.

[61] Phillips S., Takeda Y. An EEG/ERP study of efficient versus inefficient visual search.

[62] Aftanas L., Golosheykin S. (2005). Impact of regular meditation practice on EEG activity at rest and during evoked negative emotions. *International Journal of Neuroscience*.

[63] Brown C. A., Jones A. K. P. (2010). Meditation experience predicts less negative appraisal of pain: electrophysiological evidence for the involvement of anticipatory neural responses. *Pain*.

[64] Fredrickson B. L., Cohn M. A., Coffey K. A., Pek J., Finkel S. M. (2008). Open hearts build lives: Positive emotions, induced through Loving-kindness meditation, build consequential personal resources. *Journal of Personality and Social Psychology*.

[65] Amenedo E., Díaz F. (1999). Ageing-related changes in the processing of attended and unattended standard stimuli. *NeuroReport*.

[66] Staub B., Doignon-Camus N., Bacon É., Bonnefond A. (2014). The effects of aging on sustained attention ability: an ERP study. *Psychology and Aging*.

[67] Sobolewski A., Holt E., Kublik E., Wróbel A. (2011). Impact of meditation on emotional processing—a visual ERP study. *Neuroscience Research*.

[68] Lutz A., Greischar L. L., Perlman D. M., Davidson R. J. (2009). BOLD signal in insula is differentially related to cardiac function during compassion meditation in experts vs. novices. *NeuroImage*.

[69] Kisley M. A., Wood S., Burrows C. L. (2007). Looking at the sunny side of life: age-related change in an event-related potential measure of the negativity bias. *Psychological Science*.

[70] Mather M., Carstensen L. L. (2005). Aging and motivated cognition: the positivity effect in attention and memory. *Trends in Cognitive Sciences*.

