# Supplementary material for: Mindfulness Trait Predicts Neurophysiological Reactivity Associated with Negativity Bias: An ERP Study
Source: Evid Based Complement Alternat Med. 2015 Jun 1;2015:212368. doi: 10.1155/2015/212368 (PMC4466385; doi:10.1155/2015/212368)
Supplement: Supplementary file 1 — Similar correlations as presented in Table 4(a), by using the original P2 and LPP waveforms for the three categories of pictures rather than the contrasts among them, were also calculated for reference. However, no significant result was found. [file 212368.f1.docx]

**Supplementary Data**

**Table S1.** Correlations (*r*) between age, mindfulness (CAMS-R) and mean amplitudes of the original waveforms for the three categories of pictures (Pos, Neg, Neu) at P2 (with three locations: frontal, frontocentral, central) and LPP (with four locations: frontocentral, central, centroparietal, parietal)

|  | P2 | | |  | LPP | | |
| --- | --- | --- | --- | --- | --- | --- | --- |
|  | Pos | Neg | Neu |  | Pos | Neg | Neu |
| Age | .369 | .205 | .164 |  | .306 | .318 | .129 |
| CAMS-R | -.042 | -.160 | -.164 |  | -.141 | -.174 | -.243 |
|  | P2 – Frontocentral | | |  | LPP - Central | | |
|  | Pos | Neg | Neu |  | Pos | Neg | Neu |
| Age | .453 | .460 | .338 |  | .274 | .301 | .149 |
| CAMS-R | .244 | .115 | .170 |  | .092 | -.025 | -.042 |
|  | P2 – Central | | |  | LPP - Centroparietal | | |
|  | Pos | Neg | Neu |  | Pos | Neg | Neu |
| Age | .429 | .483 | .345 |  | .150 | .179 | .157 |
| CAMS-R | .374 | .214 | .284 |  | .195 | .127 | .153 |
|  |  | | |  | LPP - Parietal | | |
|  |  | | |  | Pos | Neg | Neu |
| Age |  | | |  | .019 | .016 | .074 |
| CAMS-R |  | | |  | .286 | .252 | .296 |
| Note: Note: Pos = positive; Neg = negative; Neu = neutral; CAMS-R = Cognitive and Affective Mindfulness Scale Revised  ^*^p < .05, ^**^p < .01, ^***^p < .001 (uncorrected) | | | | | | | |
